# Supplementary material for: Treatments and Outcomes in Stage I Extranodal Marginal Zone Lymphoma in the United States
Source: Cancers (Basel). 2021 Apr 9;13(8):1803. doi: 10.3390/cancers13081803 (PMC8069638; doi:10.3390/cancers13081803)
Supplement: Supplementary file 1 [file cancers-13-01803-s001.pdf]

cancers

Article

Treatments and Outcomes in Stage I Extranodal Marginal Zone Lymphoma in the United States

Juan Pablo Alderuccio [1], Jorge A. Florindez [2], Isildinha M. Reis [3,4], Wei Zhao [4] and Izidore S. Lossos [1,5,*]

[1] Department of Medicine, Division of Hematology, Sylvester Comprehensive Cancer Center, University of Miami Miller School of Medicine, Miami, FL, USA; jalderuccio@med.miami.edu
[2] Department of Medicine, Division of Hospital Medicine, University of Miami Miller School of Medicine, Miami, FL, USA; jorgeflorindez@med.miami.edu
[3] Department of Public Health Science; ireis@med.miami.edu
[4] Sylvester Biostatistics and Bioinformatics Core Resource, Sylvester Comprehensive Cancer Center, University of Miami Miller School of Medicine, Miami, FL, USA; wzhao2@med.miami.edu
[5] Department of Molecular and Cellular Pharmacology, Sylvester Comprehensive Cancer Center, University of Miami Miller School of Medicine, Miami, FL, USA
* Correspondence: ilossos@med.miami.edu;Tel.: +305-243-4785; Fax: +305-243-4787

Supplementary

Table S1. Univariable and multivariable analyses for OS and LSS in stage I EMZL who were treated with surgery and radiation.

		**Overall survival (OS)** (174 deaths)				**Lymphoma-specific survival (LSS)** (40 deaths from lymphoma and 134 deaths from other cause as competing risk)			
		**Univariable**		**Multivariable**		**Univariable**		**Multivariable**	
Variable	Category	HR (95%CI)	P	HR (95%CI)	P	SHR (95%CI)	P	SHR (95%CI)	P
**Age**	<60	Reference		Reference		Reference		Reference	
	≥60	7.10 (4.65, 10.9)	<.001	7.82 (5.09, 12.0)	<.001	5.62 (2.36, 13.4)	<.001	5.32 (2.23, 12.7)	<.001
**Sex**	Female	Reference		Reference		Reference		Reference	
	Male	1.02 (0.75, 1.38)	0.899	1.27 (0.92, 1.75)	0.149	0.72 (0.37, 1.39)	0.329	0.92 (0.46, 1.83)	0.816
**Race**	Non-Hispanic White	Reference		Reference		Reference		Reference	
	Black	1.29 (0.77, 2.17)	0.332	1.77 (1.03, 3.02)	0.038	1.69 (0.67, 4.29)	0.270	1.76 (0.69, 4.47)	0.235
	Others	0.68 (0.43, 1.10)	0.115	0.72 (0.45, 1.17)	0.186	0.49 (0.15, 1.62)	0.241	0.62 (0.19, 2.08)	0.442
**Ethnicity**	Non-Hispanic	Reference		Reference		Reference		Reference	
	Hispanic	0.98 (0.61, 1.56)	0.926	1.13 (0.70, 1.83)	0.610	1.08 (0.43, 2.71)	0.869	1.38 (0.52, 3.66)	0.518
**Primary location**	Gastric	Reference		Reference		Reference		Reference	
	Skin	0.42 (0.20, 0.89)	0.024	0.48 (0.23, 1.01)	0.054	0.15 (0.04, 0.63)	0.009	0.23 (0.05, 1.03)	0.054
	Ocular adnexa	0.68 (0.36, 1.30)	0.248	0.72 (0.38, 1.39)	0.332	0.30 (0.11, 0.85)	0.023	0.38 (0.13, 1.08)	0.070
	GI non-gastric	0.22 (0.03, 1.67)	0.142	0.15 (0.02, 1.20)	0.074	0.50 (0.07, 3.83)	0.505	0.52 (0.07, 3.97)	0.529
	Salivary glands	0.96 (0.50, 1.84)	0.901	1.02 (0.53, 1.97)	0.959	0.36 (0.12, 1.06)	0.063	0.44 (0.15, 1.26)	0.126
	Lungs	0.42 (0.12, 1.53)	0.190	0.47 (0.13, 1.71)	0.252	NE		NE	
	Breasts	0.98 (0.42, 2.31)	0.964	1.17 (0.49, 2.79)	0.731	0.80 (0.23, 2.86)	0.737	1.08 (0.29, 3.96)	0.910
	Oral	0.71 (0.27, 1.82)	0.472	0.46 (0.18, 1.21)	0.117	0.24 (0.03, 2.03)	0.192	0.24 (0.03, 1.83)	0.167
	Thyroid	0.34 (0.12, 0.91)	0.031	0.31 (0.11, 0.85)	0.023	0.28 (0.05, 1.48)	0.134	0.36 (0.07, 2.00)	0.244
	Others	0.64 (0.26, 1.59)	0.335	0.53 (0.21, 1.33)	0.176	0.72 (0.20, 2.60)	0.613	0.70 (0.20, 2.46)	0.576
**DLBCL transformation**	No	Reference		Reference		Reference		Reference	

		**Overall survival (OS)** (174 deaths)				**Lymphoma-specific survival (LSS)** (40 deaths from lymphoma and 134 deaths from other cause as competing risk)			
		**Univariable**		**Multivariable**		**Univariable**		**Multivariable**	
Variable	Category	HR (95%CI)	P	HR (95%CI)	P	SHR (95%CI)	P	SHR (95%CI)	P
	Yes	0.98 (0.31, 3.08)	0.979	0.57 (0.18, 1.80)	0.337	4.93 (1.74, 14.0)	0.003	2.80 (1.13, 6.95)	0.027

HR: hazard ratio. SHR: subdistribution hazard ratio. 95% CI: 95% confidence interval. HGT: High grade transformation.

Table 2. Non lymphoma-related causes of death.

	N	%
**Cardiovascular diseases**	499	32.0
**Respiratory diseases**	192	12.3
**Gastrointestinal-Liver diseases**	107	6.9
**Infectious diseases**	98	6.3
**Cerebrovascular disease**	83	5.3
**Alzheimer disease**	65	4.2
**Accidents-Adverse effects**	46	3.3
**Renal diseases**	37	2.4
**Other malignancies**	19	1.2
**Other causes**	412	26.4

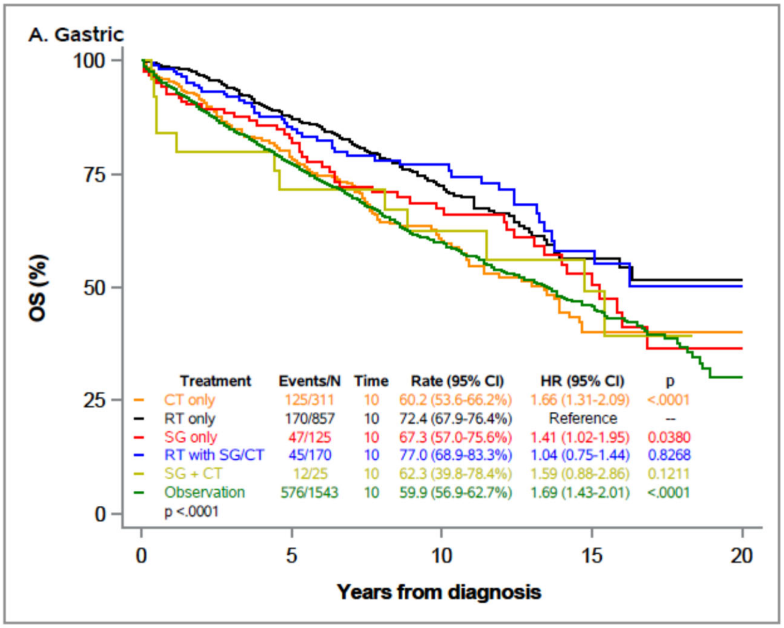

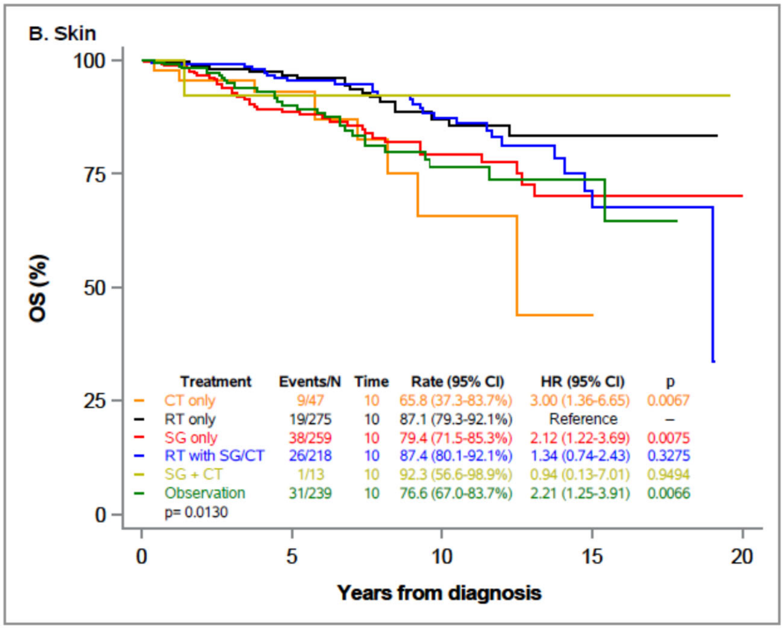

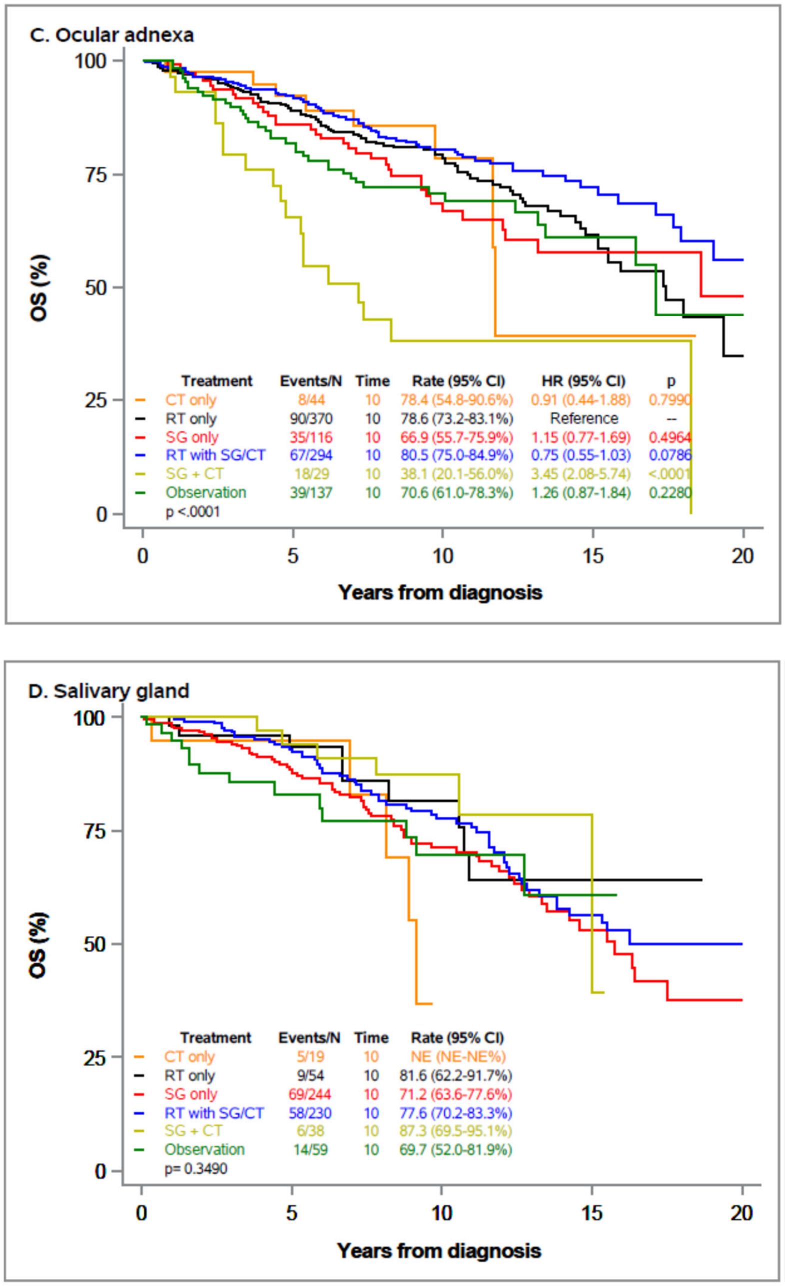

Figure 1. Overall survival (OS) by treatment strategy within EMZL primary location subgroups: Gastric (**A**), Skin (**B**), ocular adnexa (**C**), salivary gland (**D**), and lung (**E**). Note: When there was a significant effect of treatment modality on OS (overall p<0.05), plots display estimated hazard ratios and corresponding p-values from Cox regression univariable models.
